# Special Issue: Artificial Intelligence in Advanced Medical Imaging

**DOI:** 10.3390/bioengineering11121229

**Published:** 2024-12-05

**Authors:** Huang Jia, Qingliang Jiao, Ming Liu

**Affiliations:** 1 Beijing Key Lab of Nanophotonics & Ultrafine Optoelec-Tronic Systems, and School of Physics, Beijing Institute of Technology, Beijing 100081, China; hjjiayou123@163.com; 2 Beijing Key Laboratory for Precision Optoelectronic Measurement Instrument and Technology, School of Optics and Photonics, Beijing Institute of Technology, Beijing 100081, China; bit411liu@bit.edu.cn

## 1. Introduction

Medical imaging is of great significance in modern medicine and is a crucial part of medical diagnosis. Doctors can use imaging techniques such as X-ray [1], CT [2], MRI [3], and ultrasound [4] to obtain internal images of the human body. In the early stage of disease diagnosis, medical imaging can detect hidden lesions early on [5], such as tumors, increasing the possibility of curing patients. In surgical planning, effective features [6] are segmented from medical images to provide surgeons with information on the location and size of the lesion and its relationship with surrounding tissues, helping to plan a safe and effective surgical path and reducing surgical risks and complications. During the treatment process, medical imaging can monitor the treatment effect in real time and provide a basis for adjusting the treatment plan.

Deep learning [7,8,9,10] is widely used in the field of medical imaging [11]. It can handle massive medical image data [12] combined with image restoration [13], denoising [14], and other processing techniques to mine valuable information. Its image classification [15] ability can accurately distinguish between normal and diseased tissue images, helping doctors screen key cases. In image segmentation [16], it can precisely depict the boundaries of organs and lesions [17], which is of great significance for surgical planning and radiotherapy positioning. In addition, by analyzing time-series medical images, it can predict disease development and provide a basis for doctors to take preventive measures. Deep learning tools are trained with a large amount of labeled data [18], constantly improving their diagnostic accuracy and reliability [19], which can relieve the burden on doctors, promote the development of medical imaging technology, and improve the efficiency and quality of medical diagnosis [20].

We first introduce some marvelous studies about artificial intelligence in advanced medical imaging. Then, we review the work published in this Special Issue. Finally, we provide a brief summary and recommend our new Special Issue.

## 2. Related Work

In this section, we briefly introduce many excellent studies related to the scope of this Special Issue. Specifically, we introduce medical image processing, a method based on deep learning, and the multi-modality medical–biomedical image fusion method.

### 2.1. Medical Image Processing

Medical image processing can be formulated as an image-to-image transformation problem, where deep learning models are used to determine the high-dimensional nonlinear relationships between image inputs and outputs. Scholars have designed many different network architectures to solve the problem revolving around the following key question: “how can we learn this relationship effectively”?

The convolutional neural network (CNN) is a popular backbone network that has been used for medical image denoising [21,22], enhancement [23,24,25], and super resolution [26,27,28,29]. For example, the authors of [23] reported a residual network-based method for low-dose CT image texture enhancement. The authors of [28] also used a residual network for parallel MRI reconstruction. This method utilizes the correlation between the real and imaginary parts of an MRI image and considers the relevant term of k-space data, which can automatically capture the MR image correlation across channels. The authors of [30] proposed a network architecture with a residual network, attention mechanism, and U-net for CT image reconstruction. Zhang et al. [31] designed a squeeze attention module using the global spatial information of the input and obtained global descriptors for MRI super-resolution.

The Transformer, which is a backbone network in natural language processing, has recently gained widespread interest due to its powerful global information modeling ability in computer vision [32]. Zou et al. [33] designed a multi-scale deformable Transformer to capture and fuse significant auxiliary information, and it can improve the quality of knee MR images. Swin-Transformer was proven by Wu et al. [34] to have high performance in MRI reconstruction, and it is also used for super-resolution CT imaging [35]. To reduce the complexity of the Transformer, Korkmaz et al. [36] used cross-attention Transformers, which can maintain linear complexity regarding the feature map size in MRI reconstruction. To combine local information, the authors of [37] reported a hybrid CNN–Transformer network for ultrasound image segmentation, where the CNN can learn the local information, and the Transformer can exact global features.

In addition to the CNN and Transformer, the multilayer perceptron (MLP) [38] also has potential as a backbone network in biomedical image processing applications. Pan et al. [39] proved that the MLP-Mixer is a powerful tool for CT image segmentation. However, MLP still needs to be developed for application in medical images.

### 2.2. Learning Method Based on Deep Learning

Deep learning is a typical data-driven method which requires a large amount of data to learn the relationship between the input and output. However, it is unrealistic to collect a large amount of paired data for medical image processing. To solve this problem, many learning methods are applied in medical image processing.

It is worth noting that the image denoising task is especially different. Many studies on self-supervised learning have proven that denoising networks can be trained without clean references. Lehtinen et al. introduced a pioneering network named Noise2Noise [40]. They trained the network with a noisy image as the input and another noisy image as the output, where the two noisy images are independent samples of the same underlying ground truth. Many self-supervision medical image denoising methods were developed based on their study. Wu et al. [41] used the Noise2Noise as the image prior, and the network weights are fine-tuned with the iterative. Yuan et al. designed the half2half model [42], which can reduce noise in CT images. June et al. [43] developed a deep network based on Noise2Noise to improve the quality of MR images.

Self-supervision-based methods are infrequently used for other medical image processing tasks. Semi-supervision-based methods are applied in medical image segmentation. For example, Shi et al. [44] developed a semi-supervised segmentation method based on uncertainty estimation and a separate self-training strategy and achieved superior performance in CT and MR image segmentation. Lei et al. [45] used the adversarial consistency training strategy to determine the nonlinear relationships between labeled and unlabeled data and designed a bidirectional attention model based on dynamic convolution for medical image segmentation. Voulodimos et al. [46] used the few-shot learning strategy and U-net for CT regarding COVID-19 infected area segmentation. Furthermore, momentum contrastive learning also demonstrates high performance in COVID-19 infected area segmentation [47].

### 2.3. Multi-Modality Medical Image Fusion

Generally, it is impossible to extract all required information from a single imaging modality, which impacts clinical precision and efficiency. Hence, multi-modal medical image fusion is reliable for clinical usage because it can combine information from various modalities in a single image.

Liang et al. [48] developed the multi-layer concatenation fusion network, which connects the feature maps of each CNN layer with the input map, and they used down-sampling to supplement the spatial information. Zhao et al. [49] used a generative adversarial network to implement medical image fusion, and they applied a dense block to refine intermediate layer maps, which can prevent information loss. Zhang et al. [50] designed a self-supervised framework based on contrastive auto-encoding and convolution information exchange for a CT and MR image fusion task. Wang et al. [51] proved that the weakly supervised learning frame is also a powerful tool for medical image fusion. Lakshmi et al. [52] proposed an adaptive MRI-PET image fusion method; they used a residual network to enhance the resolution of the PET image, and adaptive total variation was used to decompose the input images. Xiao et al. [53] reported a Siamese pyramid network for PET and CT image fusion, and they used structure similarity and the L1 norm to build the loss function. Medical image fusion based on deep learning has been widely developed; see, for example [54,55].

## 3. Special Issue Article

The Special Issue titled “Artificial Intelligence in Advanced Medical Imaging” contains 11 articles (as of 30 June 2023).

Rahman et al. [56] proposed a CT image segmentation method for livers and tumors, and they combined the U-net and a residual network to segment the liver and assess the region of interest (ROI).

The red dot system uses expertise in the identification of anomalies to assist radiologists in distinguishing radiological abnormalities and managing them before the radiologist report is sent. Ammar et al. [57] explored whether radiographer reports are necessary and discussed whether any benefits can be highlighted to encourage health authorities worldwide to allow radiographers to write clinical reports.

Liu et al. [58] proposed an end-to-end AI-driven automatic kidney and renal mass diagnosis framework to identify abnormal areas and diagnose the histological subtypes of renal cell carcinoma.

Yossra et al. [59] developed a framework to implement the Internet of Things (IoT) and deep learning to identify lung cancer. This method deploys a DL process with a multi-layered non-local Bayes model to manage the process of early diagnosis. The Internet of Medical Things can be useful in determining factors that can effectively sort quality values through the use of sensors and image processing techniques.

They also identified lung cancer via deep learning IoT [60]. Lung cancer-related information is obtained from the IoT, and it is classified as benign or malignant. They also used Particle Swarm Optimization to optimize the proposed method.

Diffuse optical tomography is a powerful non-invasive tool for detecting breast cancer. Hauptman et al. proposed XGBoost [61], a deep learning-based method, to detect tumors in inhomogeneous breasts. They used genetic programming to improve the performance of the proposed method.

To detect the abdominal hemorrhage, Park et al. [62] used deep learning in CT imaging. They developed a cascade model to detect small lesions. The experiment showed that this method achieved 93.22% sensitivity and 99.60% specificity.

Deformable medical image registration is a critical task in clinical applications. Zou et al. [63] proposed a patient-specific method for deformable lung CT image registration, which decomposes large deformation fields into multi-continuous intermediate fields. In addition, it can estimate the deformation field for a trained network without directly relying on any intermediary images.

Dunn et al. [64] proved that the incremental multiple resolution residual network is useful for lung cancer subtype prediction. They believe this study can close the gap between algorithm study and clinical applications.

Qureshi et al. [65] evaluated the performance of four classical deep learning architectures, namely U-Net, U-NeXt, DeepLabV3+, and ConResNet, for extraocular muscle segmentation. They also used estimated EOM centroid locations, which are crucial for 3D biomechanical modeling and for the clinical management of strabismus, to assess the results.

For the diagnosis of Alzheimer’s disease, Illakiya et al. [66] designed the adaptive hybrid attention network, including enhanced non-local attention and coordinate attention. They also proposed an adaptive feature aggregation module to fuse local and non-local feature maps.

Sofia et al. [67] compared the performance of five different deep learning object detection models with varying architectures and trainable parameters. Their experiment showed that the YOLOv7tiny object detection model had the best mean average precision and inference time, and it was selected as the optimal model for this application.

Gu et al. [68] used deep transfer learning in medical image classification and proposed the “Real-World Feature Transfer Learning” framework. The authors used models pre-trained on large-scale general datasets, such as ImageNet, as feature extractors to classify X-ray images of pneumonia. They also carried out extensive experiments by means of converting grayscale images into RGB format through replicating grayscale channels and so on. The results show that this method performed excellently in multiple performance metrics and outperformed models trained from scratch, which strongly proved the effectiveness of transfer learning using general image data.

Zhang et al. [69] put forward an impulsive aggression prediction model by integrating physiological and facial expression information from video images, aiming to solve the deficiencies of the existing methods in predicting aggressive behaviors [69]. This model acquires the physiological parameters and facial expression information of subjects through IPPG (Imaging Photoplethysmography) technology and facial recognition technology and utilizes the random forest classification model to predict impulsive aggression. The experiments demonstrated that the accuracy rate of the model reaches 89.39%.

Nada et al. [70] used deep learning technology to automatically segment the levator ani muscle in 3D endovaginal ultrasound images. The training data were prepared through multiple preprocessing steps, and U-Net and its variant models were used for training and testing. The results show that the U-Net model performed excellently in terms of the mean Intersection over Union and Dice similarity coefficient metrics and that it is superior to the existing methods.

## 4. Conclusions and Prospect

The contributions in this Special Issue titled “Artificial Intelligence in Advanced Medical Imaging” take readers on a journey through topical research activities in deep learning and high-quality medical imaging. These include deep learning-based image processing, deep learning methods, and multi-modal medical image fusion. We hope that this Special Issue can provide readers with inspiration to further develop this field. Finally, we encourage readers to consider contributing to our new Special Issue (**Artificial Intelligence in Advanced Medical Imaging—2nd Edition**), and we look forward to obtaining further research on this topic.
